# Combined Administration of Metformin and Atorvastatin Attenuates Diabetic Cardiomyopathy by Inhibiting Inflammation, Apoptosis, and Oxidative Stress in Type 2 Diabetic Mice

**DOI:** 10.3389/fcell.2021.634900

**Published:** 2021-02-16

**Authors:** Weikun Jia, Tao Bai, Jiang Zeng, Zijing Niu, Daogui Fan, Xin Xu, Meiling Luo, Peijian Wang, Qingliang Zou, Xiaozhen Dai

**Affiliations:** ^1^Department of Cardiothoracic Surgery, The First Affiliated Hospital of Chengdu Medical College, Chengdu, China; ^2^Department of Cardiovascular Center, The First Hospital of Jilin University, Changchun, China; ^3^School of Basic Medicine, Chengdu Medical College, Chengdu, China; ^4^School of Biosciences and Technology, Chengdu Medical College, Chengdu, China; ^5^Department of Cardiology, The First Affiliated Hospital of Chengdu Medical College, Chengdu, China

**Keywords:** metformin, atorvastatin, diabetic cardiomyopathy, inflammation, apoptosis, oxidative stress

## Abstract

Diabetic cardiomyopathy (DCM), a common complication of diabetes mellitus, may eventually leads to irreversible heart failure. Metformin is the cornerstone of diabetes therapy, especially for type 2 diabetes. Statins are widely used to reduce the risk of cardiovascular diseases. In this study, we aimed to investigate whether the combined administration of metformin and atorvastatin could achieve superior protective effects on DCM and to elucidate its molecular mechanism. Here, *db/db* mice (9–10 weeks old) were randomly divided into four groups, including sterile water group (DM), metformin group (MET, 200 mg/kg/day), atorvastatin group (AVS, 10 mg/kg/day), and combination therapy group (MET + AVS). Mice were treated with different drugs via gavage once per day for 3 months. After 3 months of treatment, the pathological changes (inflammation, fibrosis, hypertrophy, and oxidative stress makers) were detected by histopathological techniques, as well as Western blotting. The H9C2 cardiomyocytes were treated with palmitate (PAL) to mimic diabetic condition. The cells were divided into control group, PAL treatment group, MET + PAL treatment group, AVS + PAL treatment group, and MET + AVS + PAL treatment group. The effects of MET and AVS on the cell viability and inflammation of H9C2 cells subjected to PAL condition were evaluated by terminal deoxynucleotidyl transferase-mediated dUTP nick-end labeling (TUNEL) assay, immunofluorescence staining, and Western blotting. Both MET and AVS prevented diabetes-induced fibrosis, hypertrophy, and inflammation. The combination therapy showed superior effects in protecting myocardial tissue against diabetes-induced injury. Mechanistically, the combination therapy significantly inhibited oxidative stress and the expression levels of inflammation-related proteins, e.g., NLRP3, caspase-1, interleukin-1β (IL-1β), Toll-like receptor 4 (TLR4), and P-p65/p65, in both cardiac tissues and H9C2 cells. TUNEL assay showed that the combination therapy significantly attenuated the apoptosis of cardiomyocytes; decreased the expression level of pro-apoptotic-related proteins, such as cleaved caspase-3 and BAX; and enhanced the expression level of anti-apoptotic protein (Bcl-2). Furthermore, the combination therapy remarkably upregulated the expression levels of 5′-AMP-activated protein kinase (AMPK) and SIRT1. Our findings indicated that the anti-inflammation and anti-apoptosis effects of the combination therapy may be related to activation of AMPK/SIRT1 signaling pathway.

## Introduction

Clinical trials showed that the prevalence of myocardial dysfunction in diabetic patients varies from 19 to 26%, and the outcomes associated with myocardial dysfunction are remarkably worse in patients with diabetes than in those without diabetes (Jia et al., 2018). Diabetic cardiomyopathy (DCM) is a descriptive terminology used to define myocardial dysfunctions in the presence of diabetes mellitus (DM) and in the absence of coronary artery disease, valvular heart disease, and other conventional risks for cardiovascular diseases (e.g., hypertension, dyslipidemia, and alcoholism) (Bai et al., 2016). As the prevalence of DM continues to rise, the DCM increases in parallel. Several mechanisms contribute to the development and progression of DCM, including systemic inflammation, mitochondrial dysfunction, myocardial interstitial fibrosis, altered intracellular signaling, autophagy, defective intracellular calcium transport of calcium, enhanced oxidative stress, micro-RNA altercations, and cardiovascular and metabolic disorders (Fang et al., 2015).

At present, several strategies are used to prevent or treat DCM. With respect to the positive effects of exercise and diet control on diabetes, they are also beneficial to prevent DCM. Metformin, a commonly used drug to treat diabetes for over 50 years, is significant to alleviate DCM. The positive effects of metformin on DCM are mainly associated with activation of 5′-AMP-activated protein kinase (AMPK) (Min et al., 2018), thereby, improving cardiac energy metabolism, as well as protecting heart against diabetic conditions. Metformin also influences DCM through modulating cardiac autophagy (Xie et al., 2011) and inhibiting myocardial inflammation (Yang et al., 2019) and coronary microangiopathy (Abdel-Hamid and Firgany, 2018). Statins are effective, lipid-lowering drugs with a satisfactory safety profile that have become the first-line therapy for patients with dyslipidemia and a cornerstone of ischemic cardiovascular prevention. Statins are pleiotropic drugs, inhibiting the biosynthesis of cholesterol, and they possess anti-inflammation and anti-oxidative effects. In previous animal experiments, statins could prevent DCM by alleviating left ventricular dysfunction and inhibiting myocardial fibrosis through anti-apoptosis and anti-inflammation pathways (Abdel-Hamid and Firgany, 2015; Al-Rasheed et al., 2017). Although the above-mentioned outcomes proved significant effects of statins on preventing or treating DCM, it is noteworthy that DCM is triggered by multiple complicated, cross-talked mechanisms. Thus, a single therapeutic strategy may fail, or it only partially prevents or treats DCM; for instance, intensive glucose control in a clinic failed to protect against heart under diabetic conditions (Group, 1998).

In the current study, we presented a combined therapy, metformin plus atorvastatin, for diabetic mice, and we attempted to indicated whether the combined therapy could possess superior protective effects on DCM than administration of a single drug.

## Materials and Methods

### Cell Culture and Interventions

Embryonic rat heart-derived H9C2 cells, purchased from the Shanghai Institute of Biochemistry and Cell Biology (Shanghai, China), were cultured in Dulbecco's modified Eagle's medium (DMEM; Gibco, Rockville, MD, USA), containing 100 U/ml of penicillin, 0.1 mg/ml of streptomycin, 25 mmol/L of D-glucose, and 10% fetal bovine serum (FBS; Gibco). Palmitate was used to treat H9C2 cells to mimic hyperlipidemia of type-2 diabetes. H9C2 cells were exposed palmitate (100 μM, Sigma-Aldrich, St. Louis, MO, USA) with or without metformin (1 mM, Sigma-Aldrich) and atorvastatin (10 μM, Sigma-Aldrich) in the form of single or combined treatment strategies. Palmitate solution was prepared as reported previously (Zhao et al., 2013). Briefly, palmitate was dissolved in 50% ethanol, heated at 70°C for 2 min, and added to 2% fatty acid-free bovine serum albumin (BSA; Sigma-Aldrich) medium as stock solution (2.5 mmol/L). Before use, the stock palmitate solution was gently rotated for 1 h at 37°C and further diluted to the required concentrations for treatment.

### Cell Counting Kit-8 Assay

The Cell Counting Kit-8 (CCK-8) assay was performed to evaluate the viability of H9C2 cells. H9C2 cells (1 × 10^4^ cells/well) were seeded into 96-well plates with 100 μl of DMEM and incubated for 24 h. Then, H9C2 cells were exposed to palmitate with or without metformin and atorvastatin for 48 h. After treatment, CCK-8 solution (10 μl/well) was added to the culture medium, and the cells were further incubated for 2 h. Finally, the absorbance value of medium was measured by a microplate enzyme-linked immunosorbent assay reader (Bio-Tek Instruments Inc., Winooski, VT, USA).

### Animal Models

Here, 9–10-week-old male *db/db* mice were purchased from the Model Animal Research Center of Nanjing University (Nanjing, China) and maintained under a 12:12-h light–dark cycle. All the animal experiments were approved by the Animal Research Committee of Chengdu Medical College (Chengdu, China). The mice were randomly assigned to four groups and exposed to the following interventions: vehicle group (DM group, *n* = 8), mice treated with sterilized water; metformin group (MT group, *n* = 8), mice treated with 200 mg/kg/day of metformin; atorvastatin group (AVS group, *n* = 8), mice treated with 10 mg/kg/day of atorvastatin; and atorvastatin/metformin combination group (MT + AVS group, *n* = 8), mice treated with combination of 200 mg/kg/day of metformin and 10 mg/kg/day of atorvastatin. Metformin hydrochloride tablets were purchased from Bristol-Myers Squibb (New York, NY, USA), and atorvastatin calcium was purchased from Pfizer (New York, NY, USA). Both drugs were dissolved in sterilized water and administrated by gastric gavage (orally) once per day for 3 months.

### Sample Collection and Preparation

After the last treatment, the body weight of mice was measured, and then, mice were euthanized. Blood sample was collected and processed to separate plasma. Heart tissues of mice were excised and weighed. Samples from the heart were cut. Some sample tissues were fixed in 4% paraformaldehyde and then embedded into paraffin for histological and immunohistochemical assays. Other samples were kept at −80°C for Western blotting.

### Biomedical Indicators

In order to assess changes in levels of glucose and lipid in *db/db* mice that received different treatments, the fasting blood glucose (FBG) level was measured using Accu-Chek Active Blood Glucose Test Strips (Roche, Indianapolis, IN, USA), and the levels of total cholesterol (TC) and triglyceride (TG) in plasma were detected according to the instructions presented by Nanjing Jiancheng Bioengineering Institute (Nanjing, China).

### Immunohistochemistry and Immunofluorescence Staining

To evaluate the pathologic changes, the paraffin-embedded heart samples were sliced into 3- to 4-mm-thick slices for hematoxylin and eosin (H&E) staining (Sigma-Aldrich). To detect heart fibrosis, tissue sections were stained with Masson's trichrome staining method.

For immunohistochemistry (IHC), tissue sections were dewaxed, hydrated, and then incubated with 1× target retrieval solution (Dako, Carpinteria, CA, USA) for antigen retrieval, followed by 3% hydrogen peroxide and 5% BSA for 30 min. The sections were incubated overnight at 4°C with the following primary antibodies: p-P65 (dilution, 1:500; #ab76302, Abcam, Cambridge, MA, USA), NLRP3 (dilution, 1:500; #ab214185, Abcam), caspase-1 (dilution, 1:500; #ab138483, Abcam), and interleukin-1β (IL-1β) (dilution, 1:300; #sc-12742, Santa Cruz Biotechnology, Dallas, TX, USA). After incubation with the primary antibodies, sections were incubated with secondary antibodies (dilution, 1:500 dilutions) for 1 h at room temperature. Finally, sections were treated with peroxidase substrate DAB (3,3-diaminobenzidine; Vector Laboratories, Burlingame, CA, USA) and counterstained with hematoxylin.

Heart sections were stained using fluorescein isothiocyanate (FITC)-conjugated wheat germ agglutinin (WGA; Invitrogen Inc., Carlsbad, CA, USA) to measure the cross-sectional area of cardiomyocytes. Images were visualized by using a fluorescence microscope (BX63; Olympus, Tokyo, Japan).

### Terminal Deoxynucleotidyl Transferase-Mediated dUTP Nick-End Labeling Assay

For apoptosis analysis of H9C2 cells exposed to palmitate, terminal deoxynucleotidyl transferase-mediated dUTP nick-end labeling (TUNEL) assay was performed with One Step TUNEL Apoptosis Assay Kit (Keygen Biotechnology Co. Ltd., Nanjing, China) according to the manufacturer's protocol. Images of TUNEL and DAPI-stained sections were obtained using a fluorescence microscope (BX63; Olympus, Tokyo, Japan). Only TUNEL- and DAPI-positive nuclei were counted as apoptotic nuclei.

### Dihydroethidium Fluorescence Staining

Dihydroethidium (DHE) staining (Molecular Probes, Eugene, OR, USA) was carried out to detect the level of reactive oxygen species (ROS) levels in H9C2 cells and frozen heart sections according to the previous description (Dai et al., 2017). To evaluate the levels of ROS in H9C2 cells, H9C2 cells were seeded into 24-well plates and exposed to palmitate with or without metformin and atorvastatin for 48 h. Then, H9C2 cells were twice rinsed with phosphate-buffered saline (PBS) and incubated with 5 μmol/L of DHE for 15 min at 37°C. After that, the fluorescent images of H9C2 cells were captured by a fluorescence microscope (BX63; Olympus, Tokyo, Japan), and the fluorescence intensity was detected by a microplate reader (SpectraMax M3, Molecular Devices Inc., Sunnyvale, CA, USA) under specific wave length conditions (excitation's wavelength = 518 nm; fluorescence's wavelength = 605 nm). The levels of ROS in heart tissues were determined as follows: left ventricles were excised from mice and immediately embedded into optimal cutting temperature (OCT) compound; the tissues were cut into 10-μm-thick sections and then were incubated with DHE (5 μmol/L) in PBS in a dark and humidified container at 37°C for 15 min. Finally, the fluorescence images were observed under a fluorescence microscope.

### Mitochondrial Membrane Potential Assay

The mitochondrial membrane potential (MMP) assay was performed using the JC-10 MMP assay kit according to the manufacturer's protocol. Briefly, H9C2 cells (5 × 10^4^ cells/ml) seeded into a 96-well clear bottom black plate (Eppendorf Co., Ltd., Hamburg, Germany) were exposed to palmitate with or without metformin and atorvastatin for 48 h. Following the incubation, JC-10 dye-loading solution (JC-10 and assay buffer A 1:100 v/v) was added (50 μl/well) to H9C2 cells. The plate was kept under dark conditions for 30 min. Afterwards, assay buffer B (50 μl/well) was added, and the fluorescence intensity was measured at 490/525 nm (green) and 540/590 nm (red) by a microplate reader (SpectraMax M3, Molecular Devices, Sunnyvale, CA, USA). The ratio of red/green fluorescence intensity was used to determine the MMP. The decrease in the ratio indicates the depolarization of mitochondrial membrane. To visualize the protective effects of metformin and atorvastatin on palmitate-mediated loss of MMP, the plate was analyzed under an inverted fluorescence microscope (Model 1X73, Olympus).

### Western Blot Assay

Western blot assay was undertaken to detect the protein expression. Heart tissues and harvested cells were lysed in ice-cold radio-immunoprecipitation (RIPA) lysis buffer (Santa Cruz Biotechnology). The protein concentration was determined using a Bradford Protein Assay kit (Bio-Rad Laboratories Inc., Hercules, CA, USA). The total proteins (30 μg per well) were separated on 10% sodium dodecyl sulfate–polyacrylamide gel electrophoresis (SDS-PAGE) and then transferred onto polyvinylidene fluoride (PVDF) membranes (Millipore, Billerica, MA, USA). The membranes were blocked in Tris-buffered saline with 5% non-fat milk and 0.5% BSA for 1 h and then incubated with primary antibodies overnight at 4°C, followed by incubation with the secondary antibodies for 1 h at room temperature after standard washing procedures. The primary antibodies against different target proteins were as follows: TXNIP (dilution, 1:1,000; #14715, Cell Signaling Technology, Danvers, MA, USA), NLRP3 (dilution, 1:1,000; #13158, Cell Signaling Technology), cleaved caspase-3 (dilution, 1:1,000, #9664, Cell Signaling Technology), caspase-1 β-actin (dilution, 1:1,000; #ab138483, Abcam, Cambridge, MA), AMPK (dilution, 1:1,000; #9158, Cell Signaling Technology), p-AMPK (dilution, 1:1,000, #2535, Cell Signaling Technology), SIRT1 (dilution, 1:1,000; #8964, Abcam, Cambridge, MA), Toll-like receptor 4 (TLR4) (dilution, 1:1,000; #sc-2930172, Santa Cruz Biotechnology), Bcl-2 (dilution, 1:1,000; #sc-73822, Santa Cruz Biotechnology), Bax (dilution, 1:1,000; #sc-7480, Santa Cruz Biotechnology), nuclear factor kappa B (NF-κB)-p65 (dilution, 1:1,000; #sc-8008, Santa Cruz Biotechnology), p-NF-κB–p65 (dilution, 1:1,000; #ab76302, Abcam), and β-actin (dilution, 1:3000; Bioss Biotechnology, Beijing, China). All horseradish peroxidase (HRP)-conjugated secondary antibodies were purchased from Bioss Biotechnology (Beijing, China). Blots were visualized with chemiluminescent HRP substrate (Millipore) and quantified with Quantity 5.2 software System (Bio-Rad Laboratories Inc.).

### Statistical Analysis

All data were presented as mean ± standard deviation (SD). Statistical analysis was performed using GraphPad Prism version 8.0 software (GraphPad Software Inc., San Jose, CA, USA) with one-way analysis of variance (ANOVA), followed by *post-hoc* multiple comparisons with the Scheffe test. *P* < 0.05 was considered statistically significant.

## Results

### Combined Administration of Metformin and Atorvastatin Improved the Viability and Survival Ability of H9C2 Cells Exposed to Palmitate

**Figure 1:**
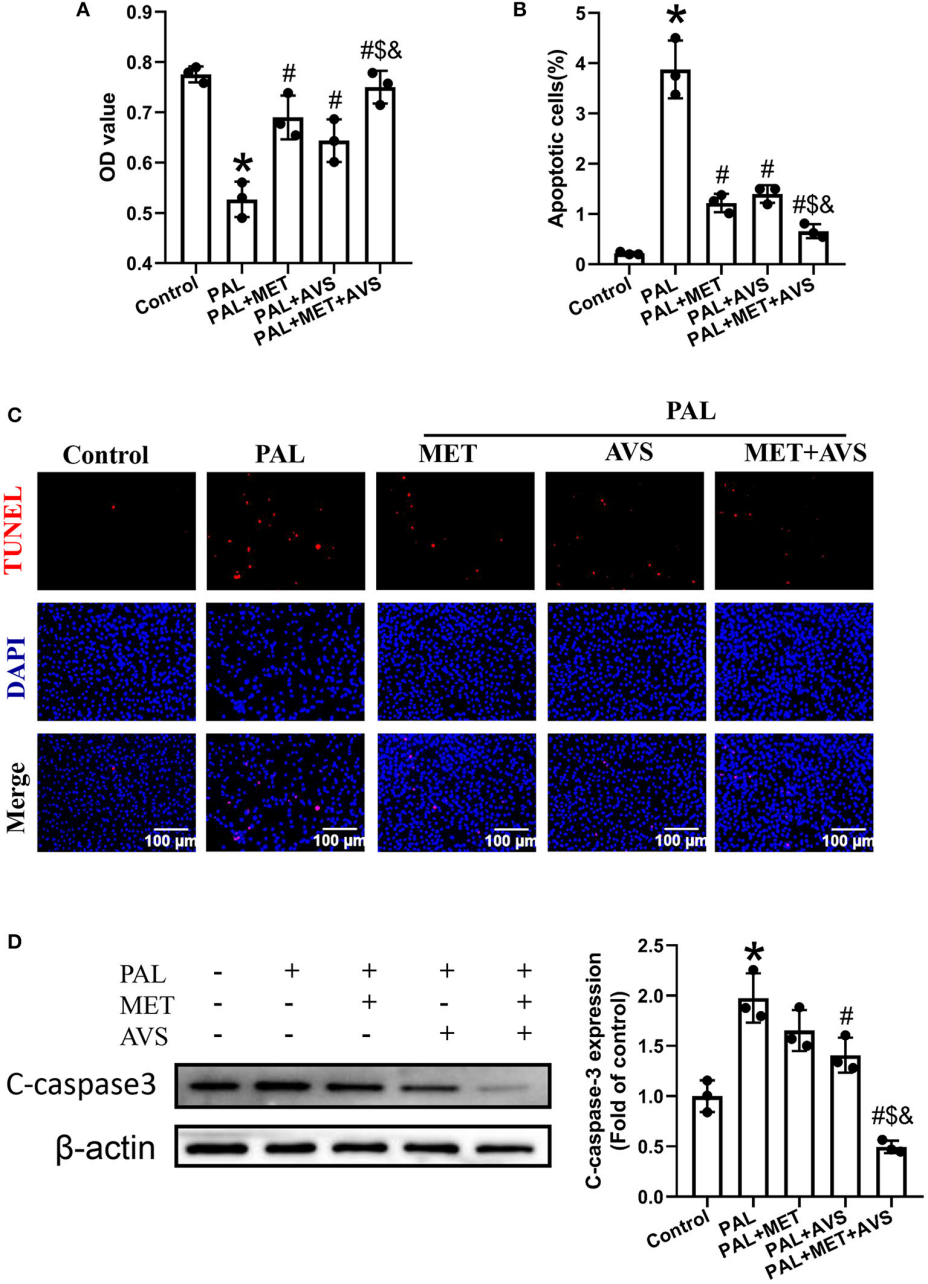
Combination therapy with metformin and atorvastatin improved the viability and survival ability of H9C2 cells exposed to palmitate. H9C2 cells exposed to palmitate (100 μM) with or without metformin (1 mM) or atorvastatin (10 μM) in the form of single or combined administration. **(A)** The cell viability of H9C2 cells exposed to palmitate was determined by Cell Counting Kit-8 (CCK-8) assay. The apoptosis H9C2 exposed to palmitate was detected by terminal deoxynucleotidyl transferase-mediated dUTP nick-end labeling (TUNEL) assay. **(B)** The apoptotic rate was calculated by TUNEL-positive cells. **(C)** The representative pictures of TUNEL staining assay. **(D)** The expression level of cleaved caspase-3 was detected by Western blotting. Three independent experiments were performed for each study. Data were presented as the means ± standard deviation (SD). ^*^P < 0.05, vs. control group; ^#^*P* < 0.05, vs. PAL group; ^$^*P* < 0.05, vs. PAL + MET group; ^&^*P* < 0.05, vs. PAL + AVS group. PAL, palmitate group; PAL + MET, metformin treatment group; PAL + AVS, atorvastatin treatment group; PAL + MET + AVS, combination treatment with metformin and atorvastatin group.

To evaluate the protective effects of metformin and atorvastatin on H9C2 cells exposed to palmitate, we investigated the viability and apoptotic rates by CCK-8 assay and TUNEL staining assay, respectively. As illustrated in Figure 1A, CCK-8 assay showed that palmitate significantly decreased the viability of H9C2 cells, both metformin and atorvastatin treatment improved the viability of H9C2 cells exposed to palmitate, and combined treatment of metformin and atorvastatin showed more significant protective efforts.

Furthermore, the apoptotic rate of H9C2 cells exposed to palmitate was determined by TUNEL staining assay (Figures 1B,C). The results showed that palmitate significantly increased the apoptosis of H9C2 cells apoptosis; besides, combination of metformin and atorvastatin could inhibit palmitate induced-apoptosis of H9C2 cells; as a result, the combined use of metformin and atorvastatin significantly decreased the apoptotic rate of H9C2 compared with treatment with either one. Subsequently, we evaluated the effects of combined therapy with metformin and atorvastatin on the expression of cleaved caspase-3, an apoptotic marker protein, by Western blot assay (Figure 1D). Consistent with the results of TUNEL assay, palmitate significantly increased the expression level of cleaved caspase-3; either metformin or atorvastatin treatment slightly decreased the expression level of cleaved caspase-3; combination treatment with metformin and atorvastatin significantly reduced the expression level of cleaved caspase-3.

### Combined Administration of Metformin and Atorvastatin Attenuated Oxidative Stress of H9C2 Cells Exposed to Palmitate

**Figure 2:**
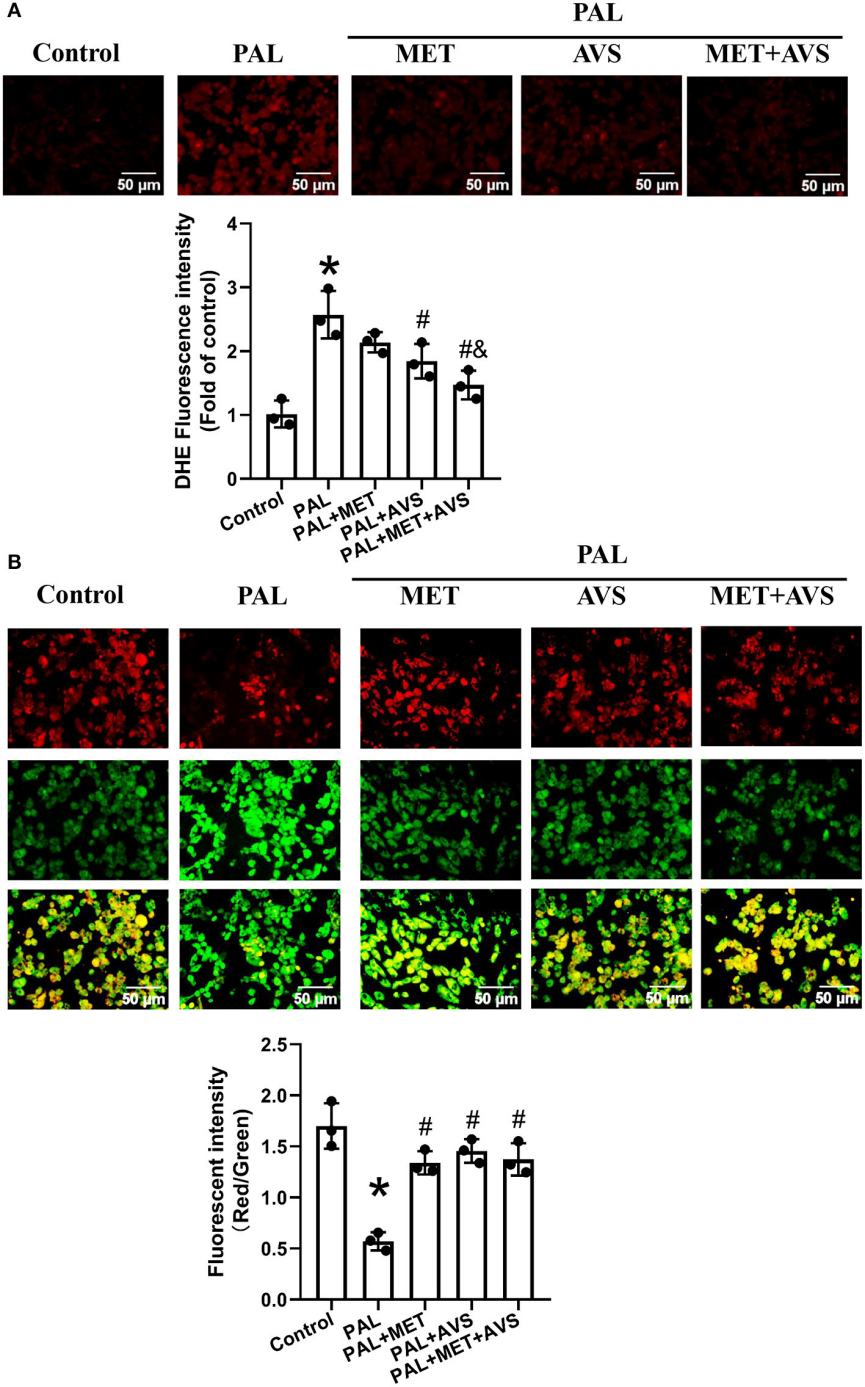
Combined administration of metformin and atorvastatin attenuated oxidative stress of H9C2 cells exposed to palmitate. **(A)** The levels of reactive oxygen species (ROS) in H9C2 cells were detected with dihydroethidium (DHE) staining; the fluorescence images were captured, and the fluorescence intensity was calculated. **(B)** The mitochondrial membrane potential (MMP) of H9C2 cells was determined by JC-10 staining; the MMP was calculated by the ratio of red-to-green fluorescence intensity. Three independent experiments were performed for each study. Data were expressed as means ± standard deviation (SD). ^*^P < 0.05, vs. control group; ^#^*P* < 0.05, vs. PAL group; ^$^*P* < 0.05, vs. PAL + MET group; ^&^*P* < 0.05, vs. PAL + AVS group.

Oxidative stress is considered as an important factor during the evolution of DCM. To determine whether combined therapy with metformin and atorvastatin could protect H9C2 cells against oxidative stress caused by palmitate, we detected the levels of intracellular ROS levels and MMP by DHE staining and JC-10 MMP assays, respectively. Expectedly, the fluorescence intensity in palmitate group was significantly higher than that in control group (Figure 2A), indicating that palmitate elevated the level of intracellular oxidative stress. In addition, metformin and atorvastatin reduced the ROS level in H9C2 cells exposed to palmitate, especially combined treatment with metformin and atorvastatin showed more valuable anti-oxidative effects (Figure 2A).

Oxidative stress may cause loss of MMP; thus, we further investigated the MMP level by MMP assay using JC-10 staining. JC-10, a cationic dye, could remain inside the healthy mitochondria to form JC-10 aggregate, generating red fluorescence at 590 nm. In unhealthy mitochondria with decreased MMP level, JC-10 exists in cytoplasm as monomers form, generating green fluorescence at 520 nm. As shown in Figure 2B, H9C2 cells in control group exhibited high red and low green fluorescence intensities. However, H9C2 cells exposed to palmitate displayed high green fluorescence intensity and low red fluorescence intensity, while treatment with metformin or atorvastatin increased the red fluorescence intensity and reduced green fluorescence intensity. Quantitative analysis of fluorescence intensity showed that palmitate significantly decreased the ratio of red/green fluorescence intensity of H9C2 cells, indicating that palmitate caused depolarization of MMP. Either metformin or atorvastatin treatment increased the ratio of red/green fluorescent intensity, and combination treatment with metformin and atorvastatin almost preserved the ratio of red/green fluorescent intensity as the control group (Figure 2B).

### Combined Administration of Metformin and Atorvastatin Inhibited NLRP3 Inflammasome in H9C2 Cells Exposed to Palmitate via Toll-Like Receptor 4/NF-κB Signaling Pathway

**Figure 3:**
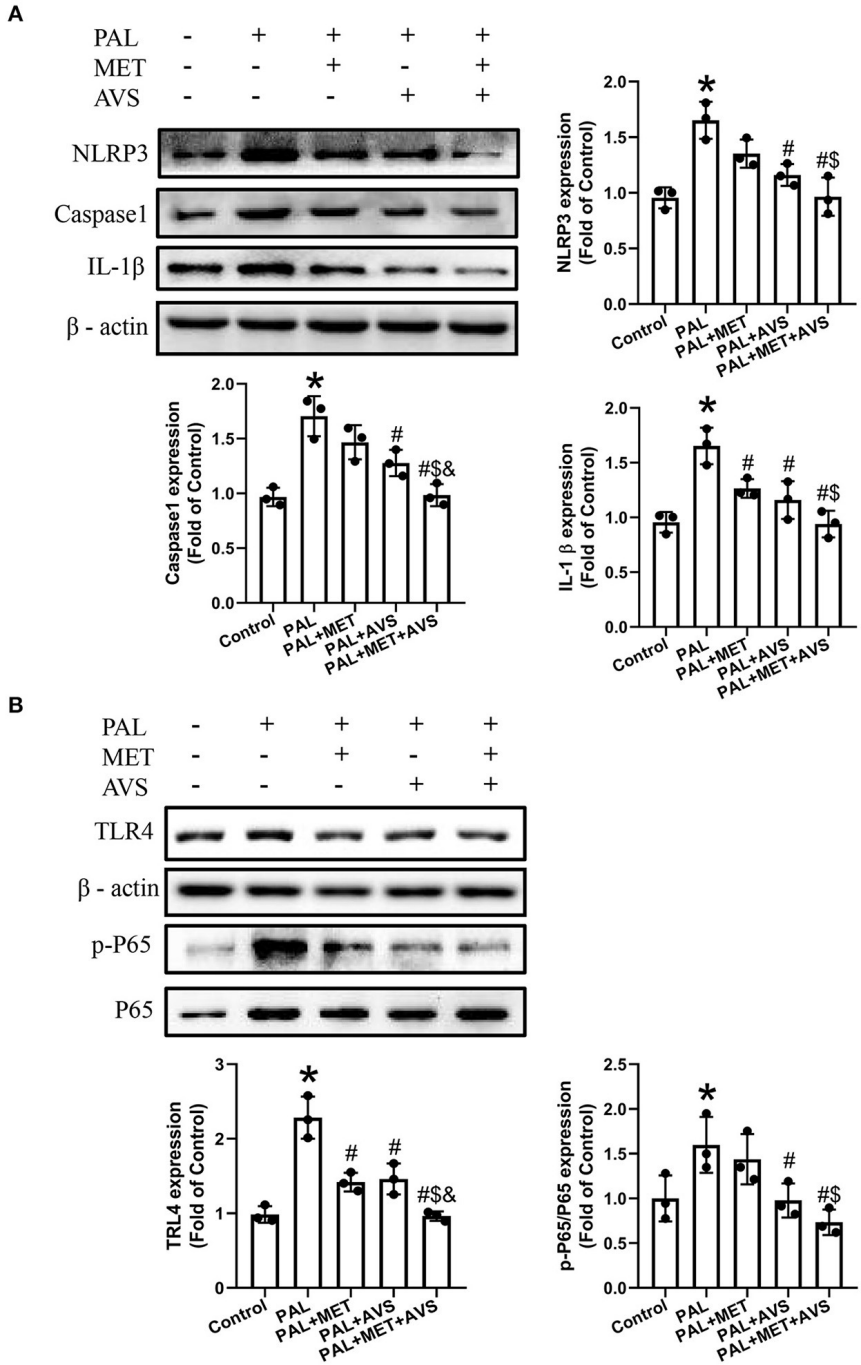
Combined administration of metformin and atorvastatin inhibited NLRP3 inflammasome in H9C2 cells exposed to palmitate via Toll-like receptor 4/nuclear factor kappa B (TLR4/NF-κB) signaling pathway. **(A)** The expression levels of NLRP3 and its downstream molecules (caspase-1 and IL-1β) were detected by Western blot assay. **(B)** The expression levels of TLR4 and NF-κB were detected by Western blot assay. Three independent experiments were performed for each study. Data were presented as the mean ± standard deviation (SD). ^*^P < 0.05, vs. control group; ^#^*P* < 0.05, vs. PAL group; ^$^*P* < 0.05, vs. PAL + MET group; ^&^*P* < 0.05, vs. PAL + AVS group.

Hyperlipidemia- and hyperglycemia-induced chronic inflammation has been proposed to contribute to DCM. Especially, NLRP3 inflammasome, expressed abundantly in cardiomyocytes, may play a pivotal role in death of myocardial cell (Luo et al., 2017). The degree of inflammation can determine cell apoptosis by affecting the expression level of caspase-1 (Thawkar and Kaur, 2019). In order to indicate whether metformin and atorvastatin could inhibit NLRP3 inflammasome activation, the expression levels of NLRP3 and its downstream molecules (caspase-1 and IL-1β) were detected by Western blot assay. The results revealed that palmitate remarkably increased the expression levels of NLRP3, caspase-1, and IL-1β (Figure 3A). Besides, a combined therapy of metformin and atorvastatin treatment significantly inhibited the protein expression as induced by palmitate. Compared with metformin and atorvastatin alone treatment, the combination treatment showed more significant therapeutic effects.

TLR4/NF-κB signaling molecular pathway plays an important role in activation of NLRP3 inflammasome (Tan et al., 2019). To determine whether combined treatment with metformin and atorvastatin treatment could inhibit NLRP3 inflammasome via TLR4/NF-κB signaling pathway, we further detected the expression level of TLR4 and NF-κB by Western blot assay. The results showed that the protein expression level of TLR4 and the phosphorylation level of NF-κB p65 were upregulated in H9C2 cells exposed to palmitate, while combined therapy with metformin and atorvastatin treatment remarkably inhibited the increase of the above-mentioned expression levels; consequently, the effects of combination treatment are more significant than either application of metformin or atorvastatin (Figure 3B).

### Combined Administration of Metformin and Atorvastatin Inhibited Diabetes-Induced Histopathological Changes in *db/db* Mice

**Figure 4:**
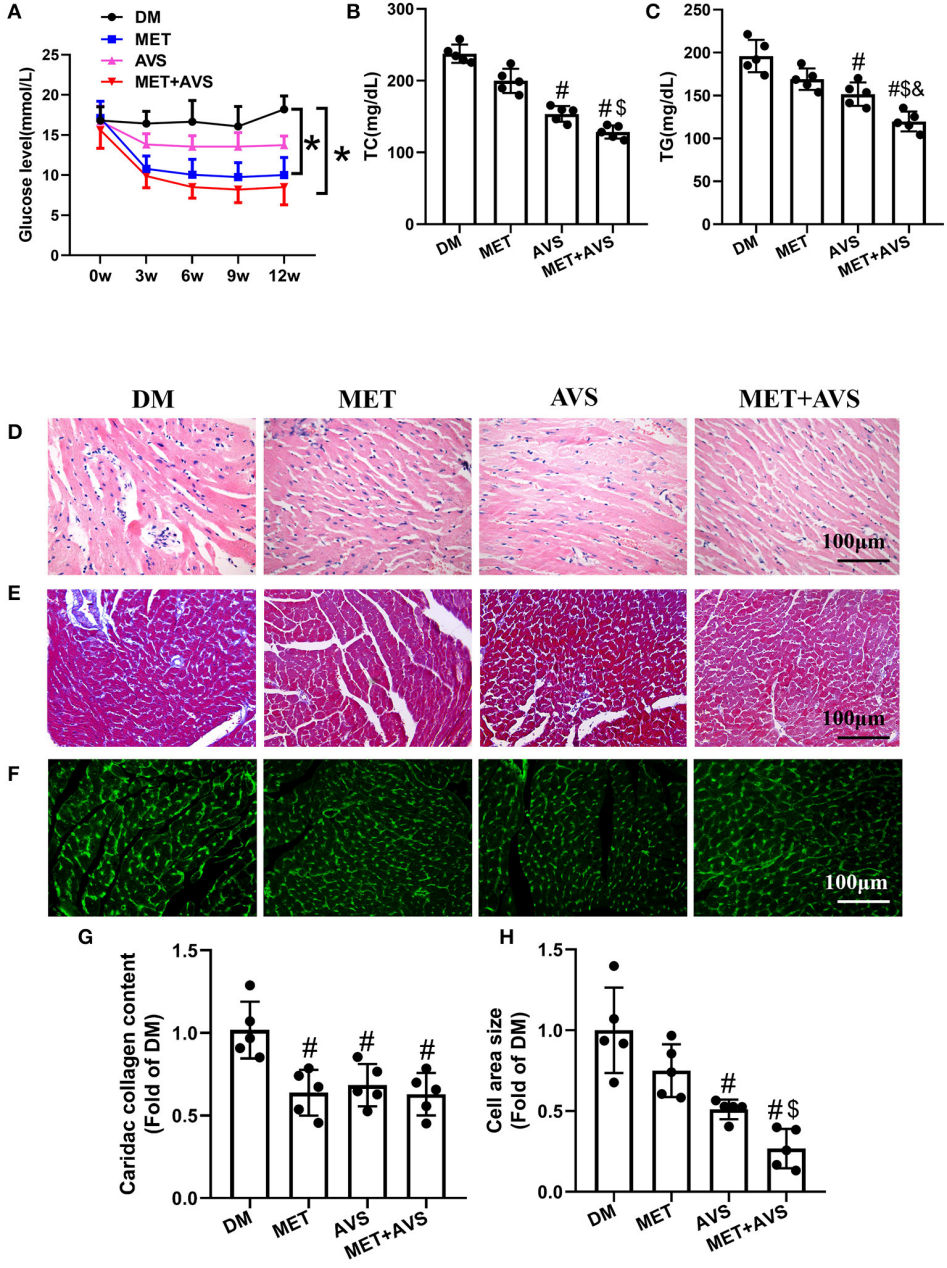
Combined administration of metformin and atorvastatin inhibited cardiac remodeling in *db/db* mice. **(A)** The fasting blood glucose level in *db/db* mice. **(B)** The level of total cholesterol (TC) in plasma. **(C)** The level of triglyceride (TG) in plasma. **(D)** Hematoxylin and eosin (H&E) staining. **(E)** Masson's trichrome staining. **(F)** Heart sections were stained using fluorescein isothiocyanate (FITC)-conjugated wheat germ agglutinin (WGA). **(G)** The quantification of cardiac collagen content. **(H)** The quantification of cross-sectional area of cardiomyocytes. Data are shown as the mean ± standard deviation (SD), *n* = 5. ^#^*P* < 0.05 vs. DM group; ^$^*P* < 0.05 vs. MET group; ^&^*P* < 0.05 vs. AVS group; DM: water treatment group, MET: metformin (200 mg/kg/day) treatment, AVS: atorvastatin group (10 mg/kg/day) treatment, MET + AVS: combination therapy group.

As displayed in Figure 4A, metformin single-treatment or combined therapy with atorvastatin and metformin significantly reduced FBG level after 3 weeks compared with the DM group, while the FBG level in atorvastatin group was slightly reduced without a significant difference compared with the DM group. Moreover, both metformin and atorvastatin treatment significantly reduced the levels of TC and TG, while the combined therapy showed more significant efforts than a single treatment (Figures 4B,C). These results indicated that combined treatment significantly improved the glucose and lipid metabolism.

Heart tissue sections of mice were stained to examine changes in the structure of heart in diabetic mice and to determine whether metformin and atorvastatin could influence the structures. H&E staining showed that the muscle structure has disorder in inflammatory cell infiltration (Figure 4D). Metformin combined with atorvastatin could effectively inhibit the morphological changes in heart muscle and decrease inflammatory cell infiltration. We further assessed the effects of metformin and atorvastatin on myocardial fibrosis by Masson's trichrome staining (Figures 4E–G). The results showed that there were more fibrosis fibers in DM group and that both metformin and atorvastatin could inhibit myocardial muscle fibrosis, while combined therapy with metformin and atorvastatin unveiled more significant effects. Moreover, staining of heart sections with FITC-conjugated WGA revealed that the cross-sectional area of cardiomyocytes is larger in DM group, and both metformin and atorvastatin treatment can decrease the cell size (Figures 4F–H).

### Combined Treatment With Metformin and Atorvastatin Inhibited Apoptosis of Cardiomyocytes and Oxidative Stress in Heart Tissues of *db/db* Mice

**Figure 5:**
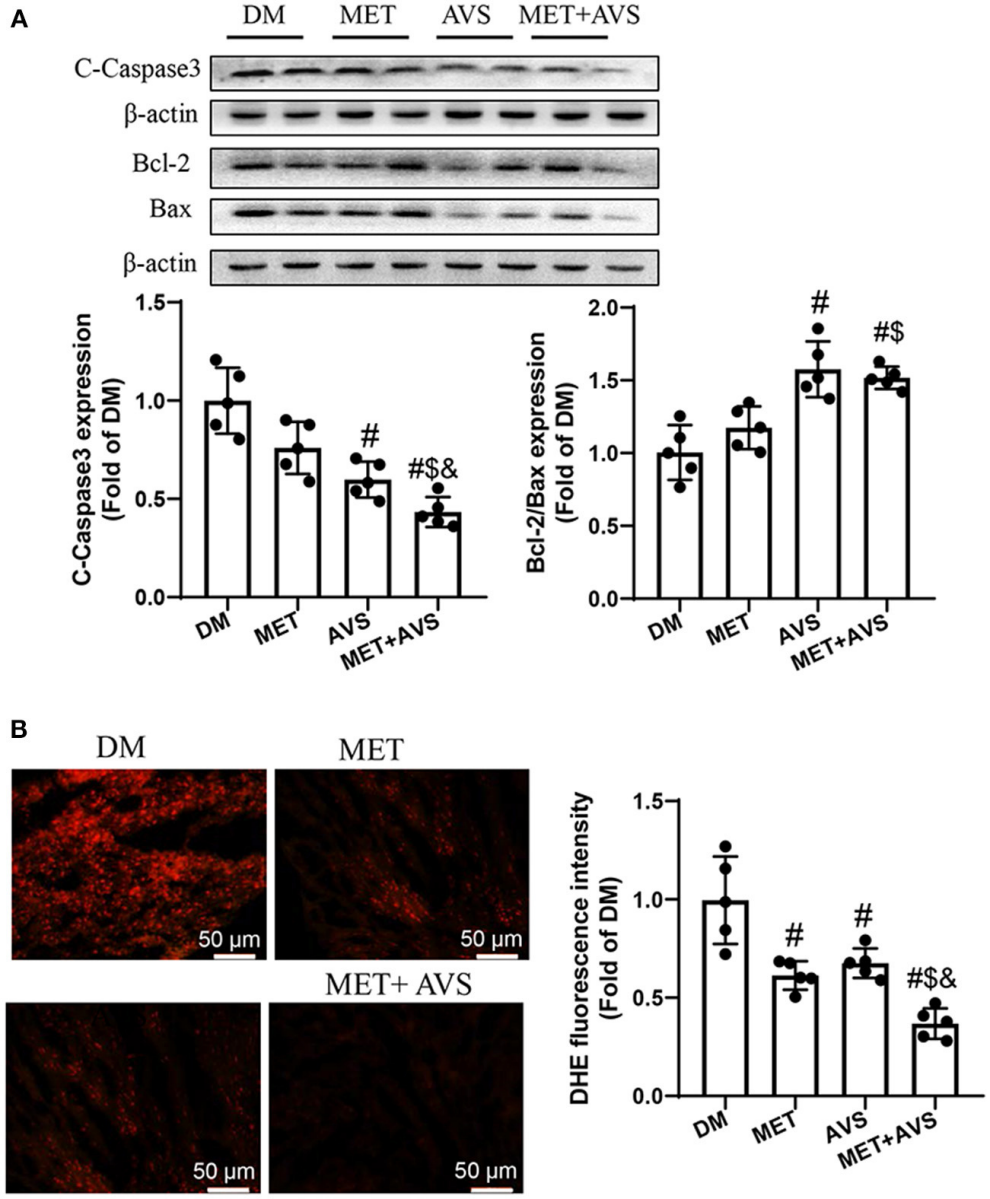
Combined treatment with metformin and atorvastatin inhibited apoptosis of cardiomyocytes and oxidative stress in heart tissues of *db/db* mice. **(A)** The expression levels of cleaved caspase-3, Bcl-2, and Bax were detected by Western blotting. **(B)** Frozen sections of heart were used to evaluate superoxide production by dihydroethidium (DHE) staining. Data were expressed as the mean ± standard deviation (SD), *n* = 5. ^#^*P* < 0.05 vs. DM group; ^$^*P* < 0.05 vs. MET group; ^&^*P* < 0.05 vs. AVS group.

The apoptosis of cardiomyocytes is an essential pathological process during the evolution of DCM. We detected the expression levels of apoptotic marker proteins (Figure 5A). The results similarly showed that the expression level of cleaved caspase-3 was higher, the ratio of Bcl-2/Bax was lower in DM group, both metformin and atorvastatin treatment can slightly decrease the expression level of cleaved caspase-3 and increase the ratio of Bcl-2/Bax, and combination treatment could markedly reduce the expression level of cleaved caspase-3 expression and increase the ratio of Bcl-2/Bax. These results demonstrated that combination treatment with metformin and atorvastatin inhibited diabetes-induced cell apoptosis.

We also detected the levels of ROS level in heart tissues stained with DHE staining (Figure 5B). The results indicated that the fluorescence intensity was high in DM group, both metformin and atorvastatin treatment decreased the fluorescence intensity, and combination treatment could more significantly decrease the fluorescence intensity. These findings suggested that combination treatment with metformin and atorvastatin could inhibit oxidative stress in heart tissues of diabetes mice.

### Combined Treatment With Metformin and Atorvastatin Inhibited Activation of NLRP3 Inflammasome in Heart Tissues of *db/db* Mice

**Figure 6:**
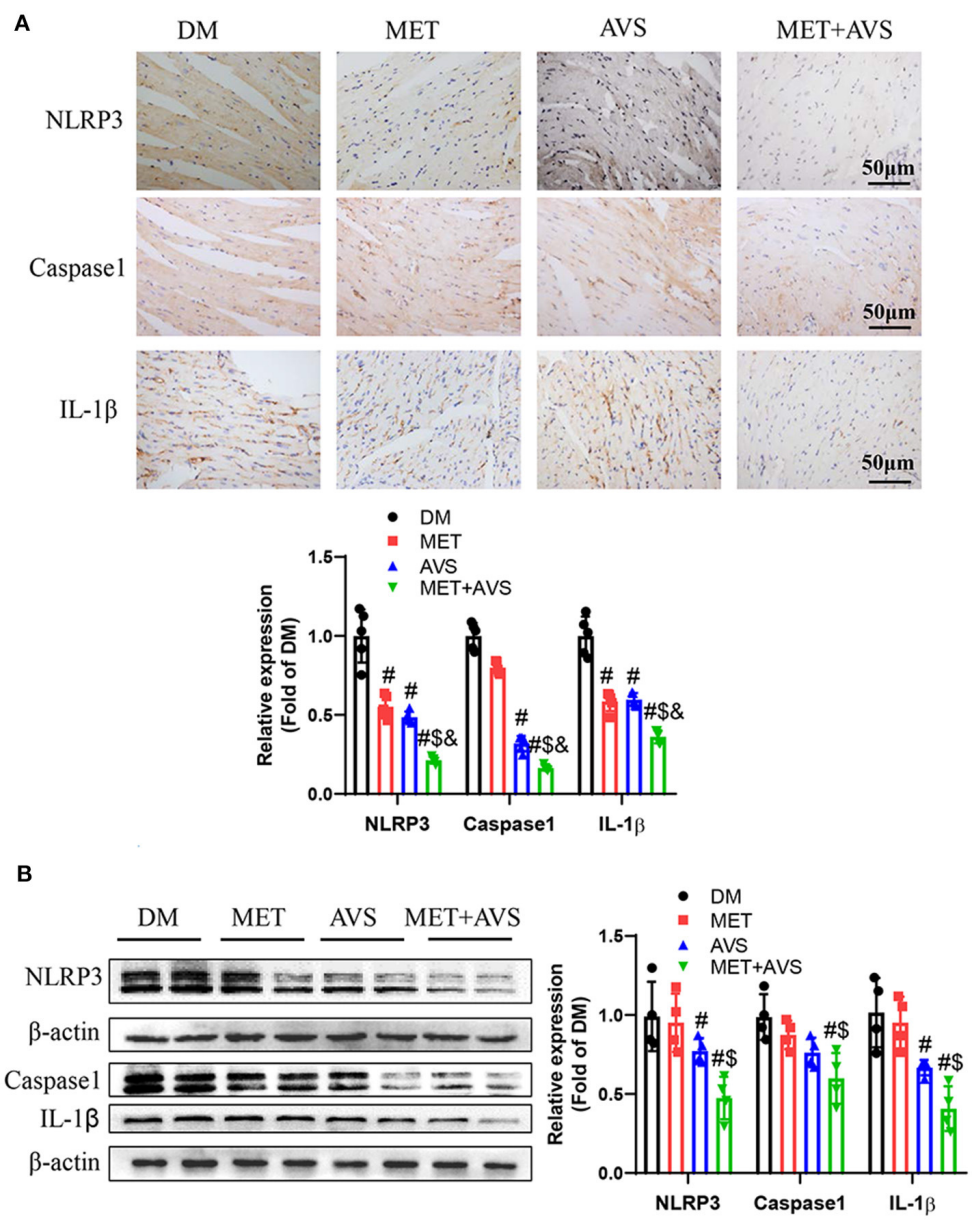
Combined treatment with metformin and atorvastatin inhibited NLRP3 inflammasome in heart tissues from *db/db* mice. **(A)** The expression levels of NLRP3, caspase-1, and IL-1β were detected by immunochemistry staining. **(B)** The expression levels of NLRP3, caspase-1, and IL-1β were detected by Western blot assay. Data were presented as the mean ± standard deviation (SD), *n* = 5. ^#^*P* < 0.05 vs. DM group; ^$^*P* < 0.05 vs. MET group; ^&^*P* < 0.05 vs. AVS group.

It was revealed that combination treatment with metformin and atorvastatin inhibited activation of NLRP3 inflammasome *in vitro* DCM model. To further confirm the anti-inflammation effects of combined therapy of metformin and atorvastatin, we detected the expression of NLRP3 inflammasome pathway-related proteins by IHC and Western blot assay. As shown in Figure 6, both metformin and atorvastatin slightly decreased the expression of NLRP3, caspase-1, and IL-1β, while the combination therapy significantly decreased the expression levels of NLRP3, caspase-1, and IL-1β expression. These results indicated that the combination therapy could inhibit activation of NLRP3 inflammasome.

**Figure 7:**
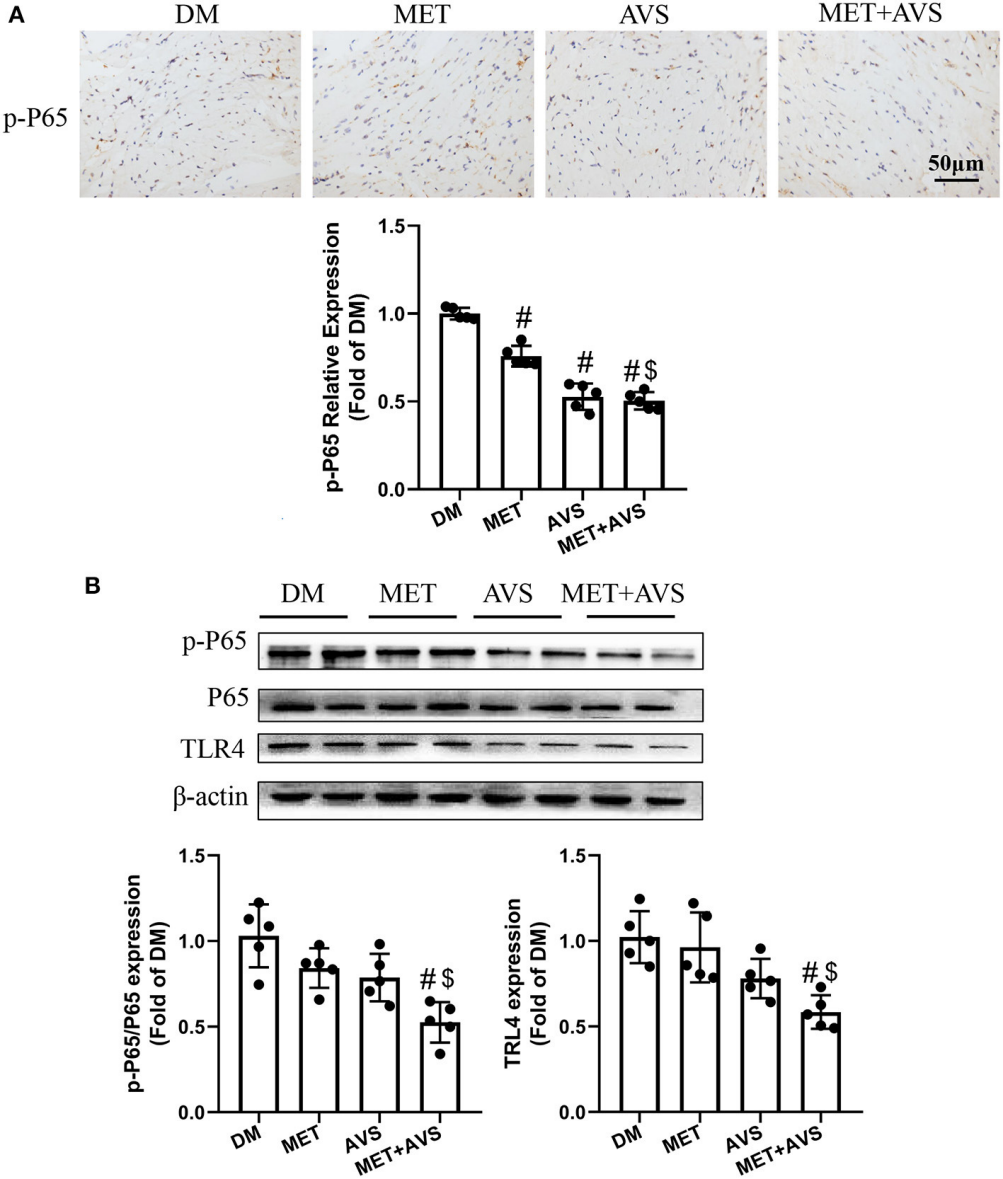
Combined treatment with metformin and atorvastatin inhibited NLRP3 inflammasome in heart tissues via TLR4/NF-κB signaling pathway. **(A)** The phosphorylation level of P65 in heart tissues was detected by immunochemistry staining. **(B)** The expression levels of TLR4 and P65 were detected by Western blot assay. Data are expressed as the mean ± standard deviation (SD), *n* = 5. ^#^*P* < 0.05 vs. DM group; ^$^*P* < 0.05 vs. MET group; ^&^*P* < 0.05 vs. AVS group.

Furthermore, we identified the expression levels of TLR4 and NF-κB. As shown in Figure 7A, the combination therapy significantly reduced the phosphorylation level of P65 in heart tissues. The results of Western blot demonstrated that the expression level of p-P65 was remarkably inhibited by the combination therapy of metformin and atorvastatin (Figure 7B). Meanwhile, the expression level of TLR4 was markedly attenuated by the combination therapy.

### Combined Treatment With Metformin and Atorvastatin Activated AMPK/SITR1 Signaling Pathway

**Figure 8:**
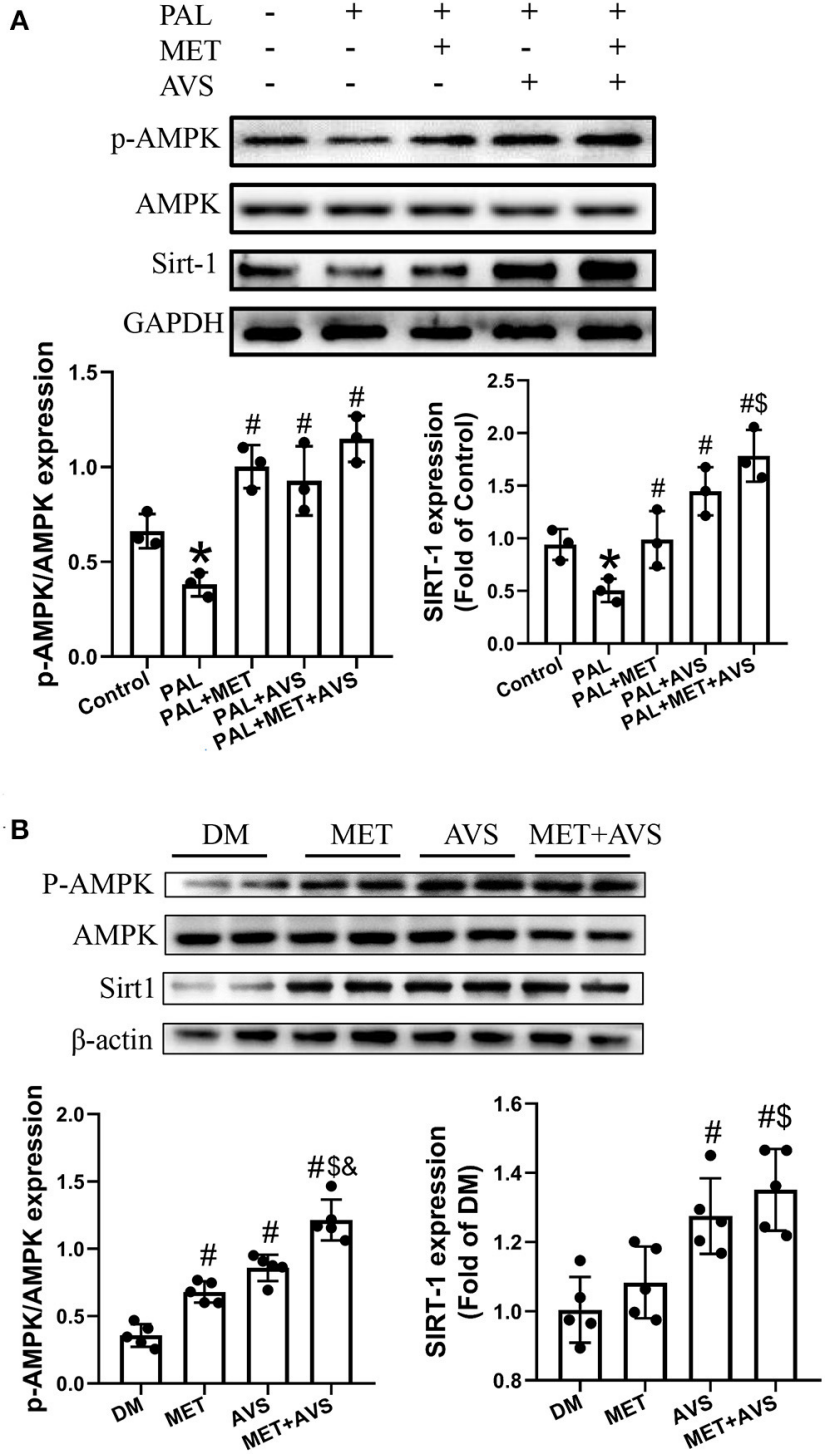
Combined treatment with metformin and atorvastatin activated 5′-AMP-activated protein kinase (AMPK)/SIRT1 signaling pathway. The expression levels of AMPK and SIRT1 in H9C2 cells **(A)** and heart tissues **(B)** were detected by Western blot assay. Three independent experiments were performed for H9C2 cells treatment. In *db/db* mice treatment, *n* = 5 in each group. Data are presented as the mean ± standard deviation (SD). ^*^P < 0.05 vs. control group; ^#^*P* < 0.05 vs. PAL group, or DM group; ^$^*P* < 0.05 vs. PAL + MET group, or MET group; ^&^*P* < 0.05 vs. PAL + AVS group, or MET + AVS group.

AMPK and SIRT1 both regulate each other and share several common target molecules. In order to explore whether combination treatment, which improved diabetic cardiomyocytes function, was correlated with the activation of AMPK/SIRT1 signaling pathway, the phosphorylation of AMPK (p-AMPK) and the expression level of SIRT1 in the H9C2 and heart tissues were assessed by Western blot. The results revealed that palmitate significantly decreased the expression level of p-AMPK and SIRT1, while combination treatment inhibited the decreased the expression level of p-AMPK and SIRT1 induced by palmitate (Figure 8A). Similarly, the combination therapy elevated the expression levels of p-AMPK and SIRT1 in diabetic heart tissues (Figure 8B). The above-mentioned findings suggested that combination treatment with metformin and atorvastatin activated AMPK/SIRT1 signaling pathway.

## Discussion

In the present study, we found that the combined use of metformin and atorvastatin achieved superior protective effects on DCM than did single administration of metformin or atorvastatin. The molecular mechanisms contributed to anti-oxidation, anti-inflammation, and anti-apoptosis. The combined use of metformin and atorvastatin may synergistically activate AMPK signaling, which is a pivotal protein in maintaining cellular homeostasis, thereby activating AMPK activator as an effective method to prevent and treat DCM.

At the early stage, the heart only showed transcriptional and metabolic altercations, including enhanced inflammation, oxidative stress, depletion of antioxidant proteins, and changes in energy metabolism. As diabetes continued, the heart showed structural changes, such as myocyte hypertrophy, apoptosis, and collagen deposition in extracellular matrix (ECM), which eventually led to massive cell death, overt cardiac hypertrophy and dilation, remarkable cardiac fibrosis, and diastolic and systolic dysfunction. As cardiac pathology in the early stage appears to be reversible (Lin et al., 2011), we may reverse damages to DCM at the early stage. In the present study, compared with single administration of metformin or atorvastatin, its combined therapy showed superior positive effects on diabetes-induced inflammation and oxidative stress.

In type 2 DM (T2DM), hyperglycemia is also accompanied with hyperlipidemia. Metformin is a widely used drug in the treatment of diabetes, possessing beneficial effects on the glucose and lipid metabolism. However, statins are a class of drugs that decrease plasma cholesterol levels and are prescribed as first choice to patients suffering from cardiovascular diseases. Previous researches have shown that low-dose (20 mg/day) atorvastatin therapy given to patients with myocardial infarction decreased insulin resistance. In the current study, the results showed that the levels of both glucose and lipid were significantly lower in combination treatment than in a single treatment. However, whether the protective effects of combination treatment are synergic remains elusive. Both metformin and statins have shown regulating glucose and lipid metabolism, whereas they could act on largely parallel pathways (van Stee et al., 2018). Previous studies demonstrated that the combination therapy with metformin and atorvastatin showed positive effects on cardiovascular hypertrophy, which are accompanied with glucose-lowering effects, lipid-lowering effects, and reduced oxidative stress (Matafome et al., 2011; Tousoulis et al., 2011; Kim et al., 2015).

Metformin exerts cardioprotective effects under diabetic conditions through multiple pathways, converging toward the activation of AMPK. A number of scholars pointed out that metformin could inhibit NLRP3 inflammasome by activating the AMPK/mTOR signaling pathway (Yang et al., 2019). SIRT1 is widely expressed in mammalian cells and has been studied in different tissues, including the liver, skeletal muscle, adipose tissue, etc. Its regulation is somewhat less clear than that of AMPK; however, a substantial body of evidence suggests that, similar to AMPK, SIRT1 responds to increases and decreases in nutrient availability. In the current study, we found that the combination therapy with metformin and atorvastatin could activate AMPK/SIRT1 signaling pathway.

Different to metformin, the use of statins in diabetes is a controversial. Although the potential detrimental effects of statin therapy on muscle and liver have been known for a long time, new concerns have emerged regarding the risk of new-onset diabetes (NOM) that often leads to discontinuation of statin discontinuation, non-adherence to therapy, or concerns correlating with initiating statin therapy. It is estimated that a 10–12% increased risk of NOM is be associated with statin therapy (Betteridge and Carmena, 2015). The mechanisms by which statins might lead to the development of NOM were not fully elucidated. The inhibition of 3-hydroxy-3-methylglutaryl coenzyme A reductase activity by statins appears to be a key mechanism (Hao et al., 2007; Han, 2018). However, statins consistently showed a protective role in the setting of DCM due to their roles of anti-inflammation, anti-oxidation, and anti-apoptosis effects (Luo et al., 2014; Carillion et al., 2017). It seems that statins may facilitate the onset of diabetes by impacting peripheral insulin sensitivity and islet β-cell function, while statins can effectively modify the promotive factors promoting DCM, including inflammation and oxidative stress, thereby protecting the heart against diabetic conditions. A recent review suggested that the benefits of statin therapy for diabetes far outweigh any real or perceived risks (Adhyaru and Jacobson, 2018), suggesting that discontinuation of statins for diabetic patients is not recommended.

In conclusion, both metformin and atorvastatin can protect DCM via their anti-inflammation and anti-apoptosis activities, and the combined administration of metformin and atorvastatin resulted in superior protective effects on DCM than a single treatment. We also found that the anti-inflammation and anti-apoptosis effects of combined therapy may be related to activation AMPK/SIRT1 signaling pathway.

## Data Availability Statement

The raw data supporting the conclusions of this article will be made available by the authors, without undue reservation.

## Ethics Statement

The animal study was reviewed and approved by Animal Research Committee of Chengdu Medical College.

## Author Contributions

XD was responsible for project administration and funding acquisition. TB was responsible for project administration and writing of the original draft. QZ, JZ, ZN, and DF performed the experiments and drafted the manuscript. QZ revised the draft and supplied additional materials. XX, ML, and PW collected and analyzed data. All authors read and approved the final manuscript.

## Conflict of Interest

The authors declare that the research was conducted in the absence of any commercial or financial relationships that could be construed as a potential conflict of interest.
